# Gaps in completion and timeliness of breast surgery and adjuvant therapy: a retrospective cohort of Haitian patients with nonmetastatic breast cancer

**DOI:** 10.1007/s10549-022-06582-8

**Published:** 2022-04-14

**Authors:** Temidayo A. Fadelu, Parsa Erfani, Joarly Lormil, Ruth Damuse, Viergela Pierre, Sarah Slater, Scott A. Triedman, Lawrence N. Shulman, Timothy R. Rebbeck

**Affiliations:** 1 Dana-Farber Cancer Institute, 450 Brookline Avenue, MA- 1B-17, Boston, MA 02215 USA; 2 Harvard Medical School, Boston, MA USA; 3 Harvard T.H. Chan School of Public Health, Boston, MA USA; 4 Hôpital Universitaire de Mirebalais, Mirebalais, Haiti; 5 Zanmi Lasante, 8A, Santo 22H,, Croix-des-Bouquet, Haiti; 6 The Warren Alpert Medical School of Brown University, Providence, RI USA; 7 Abramson Cancer Center, University of Pennsylvania, Philadelphia, PA USA

**Keywords:** Breast cancer, Low- and middle-income countries (LMICs), Breast surgery, Global surgery, Care delays, Health disparities, Global health

## Abstract

**Background:**

There are limited data on breast surgery completion rates and prevalence of care-continuum delays in breast cancer treatment programs in low-income countries.

**Methods:**

This study analyzes treatment data in a retrospective cohort of 312 female patients with non-metastatic breast cancer in Haiti. Descriptive statistics were used to summarize patient characteristics; treatments received; and treatment delays of > 12 weeks. Multivariate logistic regressions were performed to identify factors associated with receiving surgery and with treatment delays. Exploratory multivariate survival analysis examined the association between surgery delays and disease-free survival (DFS).

**Results:**

Of 312 patients, 249 (80%) completed breast surgery. The odds ratio (OR) for surgery completion for urban vs. rural dwellers was 2.15 (95% confidence interval [CI]: 1.19–3.88) and for those with locally advanced vs. early-stage disease was 0.34 (95%CI: 0.16–0.73). Among the 223 patients with evaluable surgery completion timelines, 96 (43%) experienced delays. Of the 221 patients eligible for adjuvant chemotherapy, 141 (64%) received adjuvant chemotherapy, 66 of whom (47%) experienced delays in chemotherapy initiation. Presentation in the later years of the cohort (2015–2016) was associated with lower rates of surgery completion (75% vs. 85%) and with delays in adjuvant chemotherapy initiation (OR [95%CI]: 3.25 [1.50–7.06]). Exploratory analysis revealed no association between surgical delays and DFS.

**Conclusion:**

While majority of patients obtained curative-intent surgery, nearly half experienced delays in surgery and adjuvant chemotherapy initiation. Although our study was not powered to identify an association between surgical delays and DFS, these delays may negatively impact long-term outcomes.

**Supplementary Information:**

The online version contains supplementary material available at 10.1007/s10549-022-06582-8.

## Introduction

A majority of patients with breast cancer (BC) in Haiti and other low-income countries (LICs) present with advanced disease and ultimately will succumb to their disease. Recent estimates place the BC mortality-to-incidence ratio in Haiti at 0.57, compared to 0.14 in North America [2]. With significant increases in BC incidence anticipated over the next two decades, the number of Haitians dying from BC is expected to almost double by 2040 [3]. This disproportionate burden of BC death in Haiti is, in part, driven by limited access to timely comprehensive BC care. [4–8].

Treatment of non-metastatic BC requires a multimodal approach—including surgery, radiotherapy, and systemic therapies. Unfortunately, Haiti currently has no in-country radiotherapy facilities, which means there must be optimal utilization of both surgery and chemotherapy to offer patients with BC a chance for cure. Timely access to these treatments is integral to favorable outcomes as delays are associated with an increased risk of BC recurrence and death [9–12]. A recent systematic review showed that for each 4-week delay in breast surgery, patients had an 8% increase in mortality [12]. Similarly, each 4-week delay in adjuvant chemotherapy (AC) was associated with a 9% increase in mortality [12]. Previous literature on BC care in Haiti has focused primarily on delays in presentation after onset of breast symptoms [6–8, 13]. However, little is known about the treatment experience of BC patients after initial diagnosis and how care-continuum delays in obtaining surgery or systemic therapies may affect treatment outcomes.

Our primary study objectives were threefold: first, to examine the associations between patient demographic and clinical factors, and the likelihood of completing curative-intent surgery; second, to explore associations between demographic and clinical factors, and care-continuum delays in surgery completion and AC initiation; and lastly to explore the association between surgical delays and likelihood of BC recurrence or death.

## Methods

### Study setting and population

**Fig. 1:**
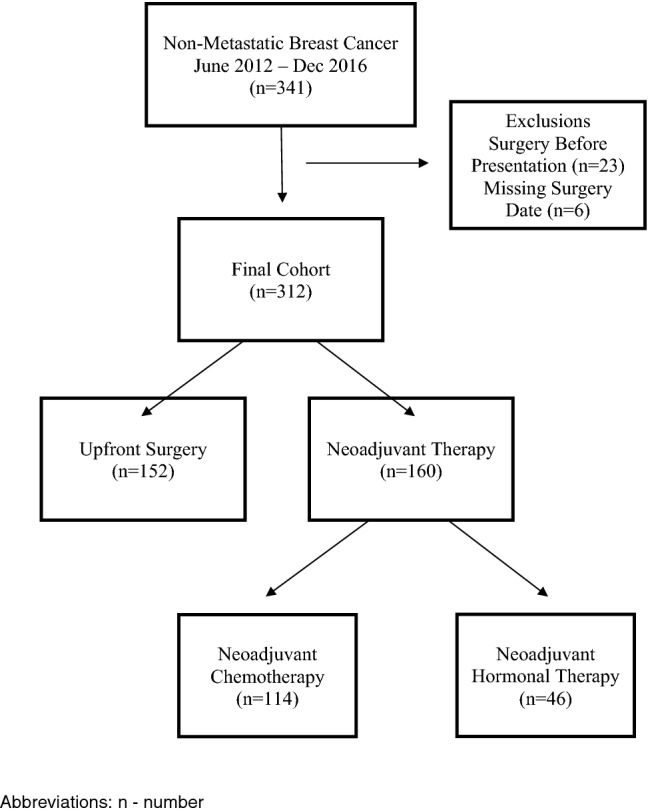
Cohort derivation

The study sample was retrospectively ascertained at Hôpital Universitaire de Mirebalais (HUM), a public, tertiary 350-bed hospital located in the Central Plateau region of Haiti [14]. The eligible study population included female patients with pathologically confirmed non-metastatic BC treated at HUM, who were diagnosed between June 2012 and December 2016 (*N* = 341). Further details of the study setting and cohort characteristics have been previously described. [15] Patients who received surgical treatment prior to presentation at HUM (*N* = 23) and those whose surgery date was unknown (*N* = 6) were excluded. The final resultant analysis cohort included 312 patients (Fig. 1). This cohort consisted of two groups, categorized by initial treatment intent: those patients who were intended to receive surgery as their first line of treatment comprised the upfront surgery group (*N* = 152) vs. patients who first received neoadjuvant therapy (NAT) with an intention to receive subsequent surgery (NAT cohort, *N* = 160). The NAT cohort was further divided into two sub-groups by type of NAT received: neoadjuvant chemotherapy (NAC) vs. neoadjuvant hormonal therapy (NAH). If a patient received both NAC and NAH, they were assigned to either the NAC or NAH cohort based on which treatment type they received last.

Research and ethical approvals were obtained from the Institutional Review Boards at Zanmi Lasante in Haiti, which governs local research at HUM, and from the Dana-Farber/Harvard Cancer Center in Boston, Massachusetts.

### Study variables and covariates

Comprehensive data were collected from the medical records on patient characteristics; diagnostic information; treatments administered; and treatment outcomes including disease recurrence and death [15]. Covariates of interest fell into the following categories: patient demographics, clinical disease characteristics, geographical information, prior medical history, and treatment details. Menopausal status was determined from recorded report of loss of periods for at least 12 months prior to presentation. For individuals with missing menopausal status (*N* = 24), patients over the age 50 years old were inferred to be post-menopausal, based on menopausal age estimates in the Caribbean region. [16] Metropolitan urban and rural residence classification was determined by the patient’s recorded home residence location. This residence location was linked to a World Bank database, which classifies residence locations as urban or rural based on population density estimates. [17] Stage at presentation was modified from the Union for International Cancer Control (UICC) TNM staging, 7^th^ edition classification; Stage I and Stage II were classified as “Early” and Stage III as “Locally Advanced.” [18] Pathologic type and grade were obtained from pathology reports based on World Health Organization classifications. [19] Estrogen receptor (ER) status was determined using guidelines from the College of American Pathologists [20]. Progesterone receptor status and human epidermal growth factor receptor 2 (HER2) amplification status were not included as covariates, as testing for both was not routinely performed in Haiti due to financial constraints and lack of access to relevant HER2-directed therapeutic agents. Except as otherwise noted, covariates with missing variables were coded as a separate “unknown” category.

### Study endpoints

The primary analyses endpoints were curative-intent breast surgery completion and time to surgery. The proportion of patients who underwent breast surgery was calculated, and the reason for not completing surgery was abstracted from the medical records. Time to surgery was assessed for the upfront surgery and NAC cohorts. For the upfront surgery cohort, time to surgery was defined as number of days from the date of initial BC consultation at HUM to the date of surgery, while for the NAC cohort, time to surgery was defined as the length of time from NAC completion to the date of surgery. Delays in surgery were defined as a time to surgery greater than 12 weeks; this threshold was based on recommendations from prior observational studies [9–12]. Patients assigned to the NAH group were omitted from the surgery delay analysis as patients commonly receive NAH until the day of surgery, and there are no consensus recommendations for duration of NAH therapy.

Time to initiation of AC was also assessed. Patients were deemed eligible for AC if they received surgery and did not previously receive doxorubicin, cyclophosphamide, or paclitaxel in the neoadjuvant setting. Time to AC was defined as the number of days from the date of surgery to the date of AC initiation. Delays in initiation of AC were also defined as greater than 12 weeks from receiving surgery [9–12]. Furthermore, we assessed disease-free survival (DFS), defined as length of time from the date of surgery to date of tumor recurrence or death from any cause.

### Statistical analysis

For baseline patient characteristics, proportions were estimated among the upfront surgery and NAT cohorts. Cohorts were compared using Chi-square test or Fisher’s exact test for categorical variables, and unpaired T test for continuous variables. Reverse Kaplan–Meier methods were used to generate cumulative incidence time to surgery curves for the upfront surgery and NAC cohorts. Patients who died before receiving surgery were censored at their death date. Time to surgery and delays in surgery were compared between the upfront surgery versus NAC cohorts using a Mann–Whitney U test and Chi-squared test, respectively. Univariate and multivariate logistic regressions were used to determine the association of covariates with receiving surgery, delays in surgery, and delays in initiation of AC. Covariates included in the multivariate models were menopausal status, metropolitan status, ER status, clinical stage, and year of presentation; these were selected based on results of univariate analyses and clinical relevance.

Kaplan–Meier analysis was also used to generate DFS curves for the patients who underwent surgery. Patients who did not have disease recurrence or die were censored at the latest date of follow-up. Log-rank tests were used to compare DFS curves between those who experienced surgical delays versus those who did not [21]. Cox-proportional hazards regression analysis was used to determine the association of DFS with clinically relevant covariates, as outlined above.

All analyses were performed using Stata/IC version 16.1 (StataCorp, College Station, TX). Reported *P* values are two-sided, and a threshold level of significance of *P* value < 0.05 was considered statistically significant. There was no adjustment of significance threshold for multiple comparisons. All data used for this analysis were abstracted between May 2018 and December 2018 and stored securely in Research Electronic Data Capture (REDCap) database. [22, 23].

## Results

### Summary of baseline characteristics

Table 1 shows a summary of the baseline characteristics for the 312 patients in the cohort. Mean age was 50.1 years (standard deviation = 11.5), and 116 patients (37%) were post-menopausal. Of the cohort, 193 patients (62%) lived in urban regions and 201 (64%) lived in the West region of Haiti, which includes the capital city of Port-au-Prince. Majority of the patients,181 (58%), had locally advanced disease. Of the 312 patients, 146 patients (47%) had initial consultation date between 2012 and 2014, while 166 patients (53%) presented between 2015 and 2016. The baseline characteristics did not differ between the upfront surgery and NAT cohorts, except for disease stage. As expected, compared to patients who underwent upfront surgery, those who received NAT had significantly higher primary T stage, regional N stage, and overall clinical stage (*p* < 0.0001).

**Table 1:** Baseline characteristics by receipt of neoadjuvant therapy

Characteristics	All *n* (%) *n* = 312	Upfront surgery *n* (%) *n* = 152	Neoadjuvant therapy *n* (%) *n* = 160	*p*-value*
Age, mean (SD)	50.1 (11.5)	50.6 (11.8)	49.6 (11.2)	0.470**
Menopausal status				
Pre-menopause	196 (63%)	93 (61%)	103 (64%)	0.560
Post-menopause	116 (37%)	59 (39%)	57 (36%)	
Home metropolitan status				
Rural	119 (38%)	58 (38%)	61 (38%)	0.995
Urban	193 (62%)	94 (62%)	99 (62%)	
Home region				
Central	65 (21%)	38 (25%)	27 (17%)	0.134
North	21 (7%)	9 (6%)	12 (8%)	
West	201 (64%)	97 (64%)	104 (65%)	
South	25 (8%)	8 (5%)	17 (11%)	
Primary T stage				
1–2	77 (25%)	51 (34%)	26 (16%)	< 0.0001
3–4	176 (56%)	61 (40%)	115 (72%)	
Unknown	59 (19%)	40 (26%)	19 (12%)	
Regional N stage				
0–1	228 (73%)	114 (75%)	114 (71%)	< 0.0001
2–3	34 (11%)	6 (4%)	28 (18%)	
Unknown	50 (16%)	32 (21%)	18 (11%)	
Final TNM stage				
Early	90 (29%)	59 (39%)	31 (19%)	< 0.0001
Locally Advanced	181 (58%)	66 (43%)	115 (72%)	
Unclear	41 (13%)	27 (18%)	14 (9%)	
ER status				
Positive	87 (28%)	42 (28%)	45 (28%)	0.970
Negative	153 (49%)	74 (49%)	79 (49%)	
Unknown	72 (23%)	36 (24%)	36 (23%)	
Biopsy histologic type				
Invasive ductal	214 (69%)	96 (63%)	118 (74%)	0.056^
Invasive lobular	11 (4%)	4 (3%)	7 (4%)	
Other	43 (14%)	23 (15%)	20 (13%)	
Unknown	44 (14%)	29 (19%)	15 (9%)	
Biopsy pathologic grade				
Well or Moderately Differentiated	73 (23%)	33 (22%)	40 (25%)	0.262
Poorly Differentiated	122 (39%)	55 (36%)	67 (42%)	
Unknown	117 (38%)	64 (42%)	53 (33%)	
Year of presentation				
2012–2014	146 (47%)	73 (48%)	73 (46%)	0.671
2015–2016	166 (53%)	79 (52%)	87 (54%)	

*Estimated by Chi-square test unless noted otherwise

**Estimated by unpaired t-test

^ Estimated by Fisher’s Exact test

Abbreviations: ER estrogen receptor, *n *number in each category, *N *regional nodal stage, SD standard deviation, T primary tumor stage

### Surgery completion

**Fig. 2:**
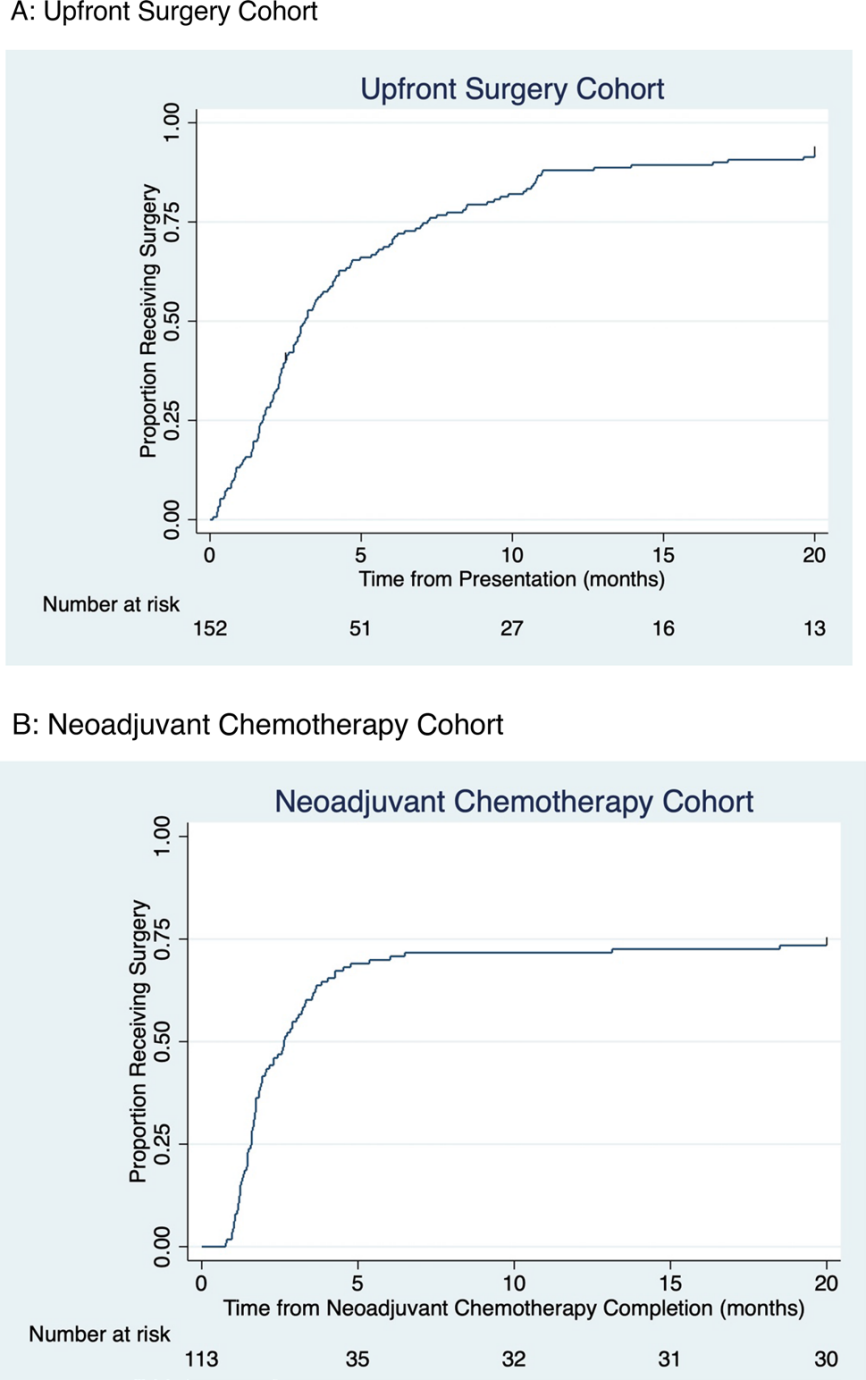
Cumulative incidence curves for time to surgery

Of the 312 patients, 249 (80%) underwent surgery (Table 2). In the upfront surgery and NAT cohorts, 139 (91%) and 110 (69%) patients underwent surgery, respectively. Of the 63 patients who did not undergo surgery, 46 were lost to follow up, 9 experienced disease progression during NAT, 4 refused surgery, and 1 passed away prior to surgery (Supplemental Table S1). Cumulative incidence curves for upfront surgery and NAT cohorts are presented in Fig. 2.

**Table 2:** Associations with surgery completion

		Univariate model		Multivariate model	
Total (*N* = 312)	Surgery completion 249 (80%) *n* (%)	Odds ratio (95% CI)	*p*-value	Odds ratio (95% CI)	*p*-value
Menopausal status					
Pre-menopause	152 (78%)	Reference			
Post-menopause	97 (84%)	1.48 (0.81–2.68)	0.198	1.53 (0.82–2.86)	0.185
Metropolitan status					
Rural	87 (73%)	Reference			
Urban	162 (84%)	1.92 (1.10–3.36)	0.022	2.15 (1.19–3.88)	0.011
ER status*					
Negative	73 (84%)	Reference			
Positive	127 (83%)	0.94 (0.46–1.91)	0.857	0.88 (0.42–1.82)	0.722
Clinical Stage**					
Early	79 (88%)	Reference			
Locally advanced	139 (77%)	0.46 (0.22–0.95)	0.035	0.34 (0.16–0.73)	0.006
Year of presentation					
2012–2014	124 (85%)	Reference			
2015–2016	125 (75%)	0.54 (0.30–0.96)	0.036	0.51 (0.28–0.94)	0.030

*Patients with unknown ER status with surgery completion = 49 (68%)

**Patients with unknown clinical stage with surgery completion = 31 (76%)

In the univariate models, receiving surgery was associated with metropolitan status, clinical stage, and year of presentation (Table 2). These associations were preserved in the multivariate logistic regression model. Patients who lived in urban areas were significantly more likely to undergo surgery compared to those in rural areas (Odds Ratio (OR) [95% Confidence Interval (CI)]: 2.15 [1.19–3.88], *p* = 0.011). Patients with locally advanced disease were significantly less likely to undergo surgery compared to women with early-stage disease (OR [95% CI]: 0.34 [0.16–0.73], *p* = 0.006). Patients who presented in 2015–2016 were also significantly less likely to undergo surgery compared to those who initiated treatment between 2012 and 2014 (OR [95% CI]: 0.51 [0.28–0.94], *p* = 0.030). Menopausal status and ER status were not associated with likelihood of completing surgery.

### Delays in surgery

Among the 139 patients in the upfront surgery cohort who received surgery, the median time from presentation to surgery was 87 days (Interquartile Range (IQR): 49–178). Among the 84 patients who completed surgery in the NAC cohort, the median time from completion of NAC to surgery was 53.5 days (IQR 40.5–89). Time to surgery was significantly greater for patients in the upfront surgery cohort compared to the NAC cohort (*p* = 0.0005) (Supplemental Table S2). Of the 223 patients who underwent surgery in the upfront surgery and NAC cohorts, 96 patients (43%) experienced surgical delays of greater than 12 weeks. Multivariate logistic regression showed no association between surgery delays and any of the examined variables (Table 3).

**Table 3:** Associations with surgical delays of greater than 12 weeks

		Univariate model		Multivariate model	
Total (*N* = 223)	Surgical delays 96 (43%) *n* (%)	Odds ratio (95% CI)	*p*-value	Odds ratio (95% CI)	*p*-value
Menopausal status					
Pre-menopause	59 (43%)	Reference			
Post-menopause	37 (44%)	1.03 (0.60–1.78)	0.910	1.05 (0.60–1.84)	0.858
Metropolitan status					
Rural	33 (40%)	Reference			
Urban	63 (45%)	1.24 (0.71–2.15)	0.445	1.24 (0.70–2.17)	0.460
ER status*					
Negative	29 (40%)	Reference			
Positive	52 (50%)	1.52 (0.83–2.78)	0.178	1.46 (0.79–2.70)	0.229
Clinical Stage**					
Early	36 (48%)	Reference			
Locally advanced	48 (40%)	0.72 (0.40–1.29)	0.273	0.72 (0.39–1.31)	0.281
Year of presentation					
2012–2014	48 (43%)				
2015–2016	48 (43%)	1.02 (0.60–1.73)	0.954	0.95 (0.54–1.66)	0.849

* Patients with unknown ER status with surgery delays = 15 (33%)

**Patients with unknown clinical stage with surgical delays = 12 (43%)

### Delays in adjuvant chemotherapy

There were 221 patients who completed surgery and were eligible for AC; of these patients, 141 (64%) received AC. Median time from surgery to initiation of AC was 83 days (IQR 65–106) (Supplemental Table S3). Of the 141 patients who received AC, 66 patients (47%) experienced delays in initiation of AC. The multivariate logistic regression model showed that patients who presented in 2015–2016 were significantly more likely to experience delays in AC initiation compared to those who presented in 2012–2014 (OR [95% CI]: 3.25 [1.50–7.06], *p* = 0.003). There was no association between delays in AC initiation and other covariates explored (Table 4).

**Table 4:** Associations with delays of greater than 12 weeks in Initiation of adjuvant chemotherapy

		Univariate model		Multivariate model	
Total (*N* = 141)	Adjuvant chemotherapy delay 66 (47%) *n* (%)	Odds ratio (95% CI)	*p*-value	Odds ratio (95% CI)	*p*-value
Menopausal status					
Pre-menopause	46 (49%)	Reference			
Post-menopause	20 (43%)	0.77 (0.38–1.57)	0.474	0.80 (0.38–1.71)	0.571
Metropolitan status					
Rural	21 (44%)	Reference			
Urban	45 (48%)	1.21 (0.60–2.43)	0.601	1.55 (0.72–3.31)	0.263
ER status*					
Negative	20 (48%)	Reference			
Positive	39 (49%)	1.07 (0.51–2.27)	0.855	1.01 (0.46–2.25)	0.977
Clinical stage**					
Early	28 (55%)	Reference			
Locally advanced	26 (37%)	0.49 (0.23–1.01)	0.054	0.55 (0.25–1.19)	0.129
Year of presentation					
2012–2014	16 (29%)	Reference			
2015–2016	50 (58%)	3.39 (1.64–6.97)	0.001	3.25 (1.50–7.06)	0.003

* Patients with unknown ER status with delays in adjuvant chemotherapy initiation = 7 (35%)

**Patients with unknown clinical stage with delays in adjuvant chemotherapy initiation = 12 (60%)

### Disease-free survival outcomes

**Fig. 3:**
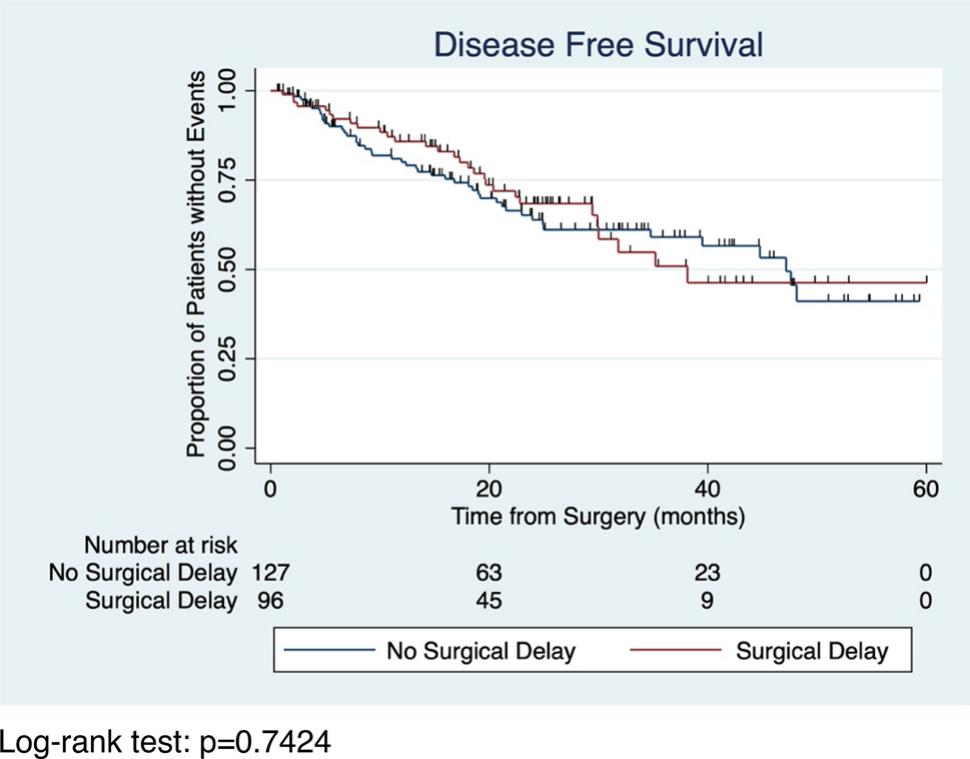
Disease-free survival by surgical delays

The median follow-up time for the cohort was 19.1 months. Of the 223 patients in the upfront surgery and NAC cohorts who received surgery, 75 patients (34%) had disease recurrence or died in the follow-up period (Table 5, Fig. 3). Multivariable Cox-Regression model showed that surgical delays were not associated with disease recurrence or death (hazard ratio (HR) [95% CI]: 1.04 (0.65–1.67), *p* = 0.866), after controlling for relevant covariates. As has been previously described in the parent cohort, compared to patients with ER-negative disease, those with ER-positive disease had longer DFS (HR [95% CI]: 0.56 (0.33–0.93), *p* = 0.026), while those with locally advanced disease had shorter DFS compared with patients with early-stage disease (HR [95% CI]: 3.05 (1.66–5.60), *p* < 0.0001) (Table 5).

**Table 5:** Multivariable cox-regression model of surgical delay and disease-free survival

		Univariate model		Multivariate model	
Total (*N* = 223)	Recurrence or death 75 (34%) *n* (%)	hazards ratio (95% CI)	*p*-value	hazards ratio (95% CI)	*p*-value
Delays					
No surgical delay	46 (36%)			Reference	
Surgical delay	29 (30%)	0.92 (0.58–1.48)	0.742	1.04 (0.65–1.67)	0.866
Menopausal status					
Pre-menopause	47 (34%)			Reference	
Post-menopause	28 (33%)	1.00 (0.64–1.56)	0.990	0.89 (0.55–1.44)	0.644
Metropolitan status					
Rural	23 (28%)				
Urban	52 (37%)	1.31 (0.81–2.13)	0.277	1.32 (0.80–2.17)	0.282
ER status*					
Negative	31 (42%)			Reference	
Positive	29 (28%)	0.49 (0.30–0.80)	0.004	0.56 (0.33–0.93)	0.026
Clinical stage**					
Early	14 (19%)			Reference	
Locally advanced	56 (47%)	3.40 (1.90–6.09)	< 0.0001	3.05 (1.66–5.60)	< 0.0001
Year of presentation					
2012–2014	52 (46%)			Reference	
2015–2016	23 (21%)	0.55 (0.34–0.90)	0.016	0.82 (0.48–1.40)	0.471

* Patients with unknown ER status with recurrence or death = 15 (33%)

** Patients with unknown clinical stage with recurrence or death = 5 (18%)

## Discussion

In this retrospective cohort of non-metastatic female BC patients treated in Haiti between 2012 and 2016, although 80% completed curative surgery, almost half them experienced surgical delays of over 12 weeks. Living in a rural area, having locally advanced disease, and presenting in later years (2015–2016) of the cohort were all associated with a higher likelihood of not receiving surgery. Approximately two thirds of patients who were eligible for AC received the treatment, and of those patients, about half experienced delays in chemotherapy initiation greater than 12 weeks. Patients who presented in 2015–2016 were more likely to experience delays in initiation of AC. While over one third of patients who received surgery had disease recurrence or died during the study follow-up period, surgical delays did not appear associated with DFS. Taken together, these findings suggest that although HUM has established access to curative treatments for a majority patients with non-metastatic BC, a substantial proportion of patients still experience significant delays during the course of their BC treatment.

The percentage of patients who received curative-intent surgery at HUM (80%) was on par with other reports from the Caribbean and other LICs; a recent study from Trinidad reported 86% surgery completion rates, while one from Rwanda reported 76% [24, 25]. However, of the 63 patients who did not receive surgery, 73% were lost to follow up. Stigma around BC and mastectomy likely contributes to this loss to follow up. [26, 27].

The factors associated with receiving surgery were largely consistent with our expectations. Patients living in rural areas were less likely to receive surgery. This finding is likely reflective of lower socioeconomic status and higher rates of poverty among the rural population in Haiti [28]. Furthermore, patients with locally advanced disease were also less likely to complete surgery which is in part due to progressive disease in some patients who received NAT. Some of these patients have such large primary tumors at diagnosis that they are unable to be to rendered surgical candidates given the limited NAT options available in Haiti. There were also higher rates of loss to follow up in this group; this further highlights the need for systematic care support structures, since patients with locally advanced disease require up to six months of NAT and frequent visits every 3 weeks prior to surgery.

Interestingly, patients who presented in the later years from 2015 to 2016 were less likely to receive surgery: 75% compared to 85% for those from 2012 to 2014. This finding was unexpected as we anticipated that maturity of the cancer program over time would lead to more streamlined care and improved care quality [29]. There are several possible explanations for this finding. HUM was established in 2013, and in addition to oncology, has also served as a national referral center for other surgery services. As patient volumes rapidly increased, there was a lag in proportionally increasing both the number of clinical staff and other care resources to meet the need. The lag likely led to stress points at the hospital and suboptimal care delivery during the later years of the cohort. For example, for most of the cohort period, the program only had one dedicated medical oncology physician provider and no dedicated oncology surgeons. These bottlenecks in oncology care resources at HUM have improved since 2016; the program now has three dedicated physicians and has established a multidisciplinary clinic attended by a dedicated surgeon and surgery residents. [29].

In addition, this study highlights substantial delays in obtaining curative-intent surgery, with over 40% of cohort having delays of over 12 weeks. The median time to surgery was 87 days for the upfront surgery cohort and 53.5 days for the NAC cohort. These times are on par with other studies in similar settings; in a review from sub-Saharan Africa, more than half of the patients had upfront surgery delays exceeding 3 months. [30] Similarly, a Rwandan cohort noted time to surgery of over 50 days among those who received NAC [25]. These times are substantially longer than those in the United States, where the median time from initial consultation to breast surgery is 29 days for those receiving upfront surgery [31]. Such surgical delays may increase rates of BC morbidity and mortality [9, 11, 12]. Suprisingly, this analysis did not identify an association between surgical delays and DFS. Likely explanations for this finding include lack of sufficient statistical power from the cohort sample size and the relatively short follow-up.

Furthermore, our analysis also identifies several gaps in AC administration. First, about one-third of patients who were eligible for AC did not receive treatment, which likely increases their risk of poor long-term outcomes [32]. Second, two-thirds of those who received AC experienced delays in chemotherapy initiation of over 12 weeks. Similar to delays in surgery, these delays can also impact on overall survival [12, 33–35]. Third, similar to the trend in surgery completion, patients who presented in later years (2015–2016) were more likely to experience delays in AC initiation. It is generally accepted that AC ought to be initiated as soon as possble after surgical healing; earlier initiation within 4 weeks is thought to provide the maximum benefit [12, 36]. While there is no absolute threshold of time following curative BC surgery where AC is no longer beneficial, 12 weeks is generally considered a substantial delay. System improvements have already been enacted that will likely reduce delays, improve care quality, and optimize AC use. These include improvement of onsite pathology services to reduce the turn-around time of breast resection pathology results; increase in clinical staffing to reduce appointment wait times; and enhanced patient education and support.

This study has several limitations. First, the size of the patient cohort, missing data, and short follow-up limit the robustness of our regression analyses. To optimize analytic power, missing variables were coded as “unknown” and included in regression analyses. Still, these limitations likely led to the lack association between surgical delays and DFS. Future analyses within this cohort after longer-term follow-up may uncover the true impact of surgery delays. Second, the 12-week definition of treatment delays represents an artificial binary cutoff. Although 12 weeks was chosen based on the cohort distribution and based on other studies’ thresholds of clinically meaningful delays, shorter delays of as little as 4 weeks may also have clinical consequences [12, 25]. While examining a range of time delays, such as 4–8, 9–12 weeks, > 12 weeks, would have been optimal, this analysis was not possible due to the limited study sample size. Nonetheless, this study does highlight both the magnitude and high prevalence of treatment delays in the Haiti BC care continuum. Lastly, DFS was reported from time of surgery, and thus, patients who did not receive surgery were excluded from the survival analysis.

This study adds to a growing body of literature that characterizes the surgical and systemic treatment care continuum for patients treated in cancer programs in LICs. These efforts are leading to the emergence of context-specific quality metrics to guide meaningful quality improvement efforts [37–39]. Ongoing efforts at HUM are focused on identifying and supporting vulnerable patients, as well as systematically tracking where patients are in the care pathway to ensure retention in care and receipt of timely care. Future studies are necessary to investigate care processes at HUM such as diagnosis turn-around, operating room availability, as well as to systematically collect patient-reported barriers. In addition, larger community and national challenges including natural disasters, earthquakes, hurricanes, pandemics, and political instability further compound patient vulnerability. These larger-scale factors may also test the resilience of the care system.

## Conclusion

The collective findings of this analysis suggest that despite multiple barriers, patients with non-metastatic BC in Haiti can access curative-intent breast surgery and systemic treatment. Significant care-continuum delays exist in surgery completion and initiation of AC, which may negatively impact patient long-term outcomes. Systematic and contextual quality improvement approaches, and comprehensive patient support system will be necessary to improve care delivery.

## Supplementary Information

Below is the link to the electronic supplementary material.

Supplementary file1 (DOCX 95 KB)

## Data Availability

The datasets used and/or analyzed during the current study are available from the corresponding author on reasonable request.

## Abbreviations

BC: Breast cancer
DFS: Disease-free survival
ER: Estrogen receptor
IQR: Interquartile Range
HR: Hazard ratio
HUM: Hôpital Universitaire de Mirebalais
LICs: Low-income countries
LMICs: Low- and middle-income countries.
NAC: Neoadjuvant chemotherapy
NAH: Neoadjuvant hormonal therapy
NAT: Neoadjuvant therapy
OR: Odds ratio
REDCap: Research Electronic Data Capture
UICC: Union for International Cancer Control
