# Supplementary material for: Gaps in completion and timeliness of breast surgery and adjuvant therapy: a retrospective cohort of Haitian patients with nonmetastatic breast cancer
Source: Breast Cancer Res Treat. 2022 Apr 14;193(3):625–35. doi: 10.1007/s10549-022-06582-8 (PMC9114044; doi:10.1007/s10549-022-06582-8)
Supplement: Supplementary file 1 — Supplementary file1 (DOCX 95 KB) [file 10549_2022_6582_MOESM1_ESM.docx]

**Supporting Information**

Supplemental Table 1. Reasons for Not Receiving Surgery

|  | **All**  **n=63** | **Upfront Surgery n=13** | **NAT**  **n=50** |
| --- | --- | --- | --- |
| Lost to follow up | 46 (73%) | 10 (77%) | 36 (72%) |
| Lack of response to NAT (disease progression) | 9 (14%) | 0 (0%) | 9 (18%) |
| Refused surgery | 4 (6%) | 1 (8%) | 3 (6%) |
| Death | 1 (2%) | 1 (8%) | 0 (0%) |
| Other | 3 (5%) | 1 (8%) | 2 (4%) |

Abbreviations: n – number, NAT – neoadjuvant therapy

Supplemental Table 2. Time to Surgery

|  | **Median**  **(days)** | **IQR** | **p value^** |
| --- | --- | --- | --- |
| Upfront Surgery cohort* (N=139) | 87 | 49-178 | 0.0005 |
| NAC cohort** (N=84) | 53.5 | 40.5-89 |  |

*Time from presentation to surgery

**Time from NAC completion date to surgery

^ Two-sample Wilcoxon rank-sum (Mann-Whitney) test (N=223)

Supplemental Table 3. Initiation of Adjuvant Chemotherapy

| No. of patients eligible for adjuvant chemotherapy | 221 |
| --- | --- |
| No. of patients receiving adjuvant chemotherapy (%) | 141 (64%) |
| Median time from surgery to initiation of adjuvant chemotherapy | 83 (65-106) |
